# Genome Sequence of *Lactobacillus johnsonii* Strain W1, Isolated from Mice

**DOI:** 10.1128/genomeA.00561-16

**Published:** 2016-06-16

**Authors:** Xiaolin Wu, Chunyan Zhao, Zhonghe Guo, Yuchong Hao, Jinghua Li, Hongyan Shi, Yanbo Sun

**Affiliations:** Department of Pathogen Biology, College of Basic Medical Sciences, Jilin University, Changchun, People’s Republic of China

## Abstract

*Lactobacillus johnsonii*, a member of the gut lactobacilli, plays an important role in normal gut functioning. Here, we report the draft genome sequence of *L. johnsonii* strain W1 isolated from ICR mice.

## GENOME ANNOUNCEMENT

*Lactobacillus johnsonii* is a Gram-positive non-spore-forming bacillus named for its capacity to produce lactic acid. *L. johnsonii* strains are frequently found in the gastrointestinal tract of several hosts and are common human and animal gut commensals (1, 2). In recent years, *L. johnsonii* strains have been subjected to extensive studies due to their probiotic activity or acting as tools for the delivery of biological active molecules, such as endolysins (3–5). Here, we describe the draft genome sequence of *L. johnsonii* strain W1 isolated from the intestinal contents of ICR mice.

The genome of *L. johnsonii* strain W1 was sequenced using paired-end sequencing with an Illumina MiSeq platform. A total of 34,929,821 reads were generated, representing an approximately 42-fold coverage of the genome. These reads were *de novo* assembled with SOAP*denovo* 2.04.r240, generating 49 contigs with an *N*_50_ of 242,274 bp. The full annotation was performed by the NCBI Prokaryotic Genome Annotation Pipeline to predict open reading frames (ORFs). The *L. johnsonii* W1 draft genome sequence has a total of 2,064,674 bp, with an average G+C content of 34.2%. It contains 1,871 predicted coding sequences (CDSs), 48 tRNAs, and one rRNA gene. Among the 1,871 predicted protein-coding sequences in the genome, 78 ORFs (4.17%) matched a hypothetical protein sequence in the public database. A phylogenetic tree based on 16S rRNA genes showed that strain W1 is most closely related to *L. johnsonii* strain KLDS 1.0734 (accession no. EU626019), and a dendrogram based on genomic Blast suggested that strain W1 is closely associated with *L. johnsonii* strain NCC 533 (6), which served as a reference genome. The strain W1 harbors several phosphotransferase systems, ABC transporters, facilitating the transportation and utilization of sugars in the gastrointestinal tract, and choloylglycine hydrolase, which is responsible for the reduced serum cholesterol concentration (7–9), suggesting its potential as a probiotic product.

### Nucleotide sequence accession numbers.

This whole-genome shotgun project has been deposited at GenBank under accession no. LSNG00000000. The version described in this paper is the ﬁrst version (LSNG00000000.1).
